# Community‐ and individual‐level correlates of HIV incidence in HPTN 071 (PopART)

**DOI:** 10.1002/jia2.26155

**Published:** 2023-08-29

**Authors:** Timothy Skalland, Helen Ayles, Peter Bock, Justin Bwalya, Kwame Shanaube, Nkatya Kasese, Michelle Dupré, Barry Kosloff, Sian Floyd, Ethan Wilson, Ayana Moore, Susan Eshleman, Sarah Fidler, Richard Hayes, Deborah Donnell, the HPTN 071 (PopART) Study Team

**Affiliations:** ^1^ Fred Hutchinson Cancer Center Seattle Washington USA; ^2^ Zambart Lusaka Zambia; ^3^ London School of Hygiene and Tropical Medicine London UK; ^4^ Desmond Tutu TB Centre Department of Paediatrics and Child Health Faculty of Medicine and Health Sciences Stellenbosch University Cape Town South Africa; ^5^ FHI 360 Durham North Carolina USA; ^6^ Johns Hopkins University School of Medicine Baltimore Maryland USA; ^7^ UK Department of Infectious Disease Faculty of Medicine, Imperial College NIHR BRC London UK

**Keywords:** PopART, HIV, incidence, cluster trial, PDV, correlates

## Abstract

**Introduction:**

Universal HIV testing and treatment aims to identify all people living with HIV and offer them treatment, decreasing the number of individuals with unsuppressed HIV and thus reducing HIV transmission. Longitudinal follow‐up of individuals with and without HIV in a cluster‐randomized trial of communities allowed for the examination of community‐ and individual‐level measures of HIV risk and HIV incidence.

**Methods:**

HPTN 071 (PopART) was a three‐arm cluster‐randomized trial conducted between 2013 and 2018 that evaluated the use of two combination HIV prevention strategies implemented at the community level to reduce HIV incidence compared to the standard of care. The trial, conducted in 21 communities in Zambia and South Africa, measured HIV incidence over 36 months in a population cohort of ∼2000 randomly selected adults per community aged 18–44. Multilevel models were used to assess the association between HIV incidence and community‐ and individual‐level socio‐demographic and behavioural risk factors, as well as prevalence of detectable virus (PDV) defined as the estimated proportion of the community with unsuppressed viral load.

**Results:**

Overall HIV incidence was 1.49/100 person‐years. Communities with less financial wealth and communities with more individuals reporting having sex partners outside of the community or two or more sexual partners had higher HIV incidence. PDV at 2 years of study was 6.8% and was strongly associated with HIV incidence: for every 50% relative reduction in community PDV, there was a 49% (95% confidence interval [CI]: 37%–58%, *p* < 0.001) relative decrease in HIV incidence. At the individual level; socio‐economic status, AUDIT score, medical male circumcision and certain sexual behaviours were associated with HIV risk.

**Conclusions:**

Using data from the PopART randomized trial, we found several associations of HIV incidence with community‐level measures reflecting the sexual behaviour and socio‐economic make‐up of each community. We also found a strong association between community PDV and HIV incidence supporting the use of PDV as a tool for monitoring progress in controlling the epidemic. Lastly, we found significant individual‐level factors of HIV risk that are generally consistent with previous HIV epidemiological research. These results have the potential to identify high high‐incidence communities, inform structural‐level interventions, and optimize individual‐level interventions for HIV prevention.

**Clinical Trial Number:**

ClinicalTrials.gov number, NCT01900977, HPTN 071 [PopArt]

## 1 INTRODUCTION

The risk of HIV acquisition varies widely between countries in the same region, between communities within the same country and between individuals within the same community. Understanding factors that influence HIV acquisition at the individual level has been an important focus of research since the beginning of the HIV epidemic, leading to the development and testing of successful interventions to reduce the risk of acquisition for individuals [1]. Individual risk is associated with direct exposure risk (e.g. sexual and injection behaviour) which is influenced by socio‐behavioural and socio‐economic factors. However, community‐level HIV risk factors that influence an individual's risk of acquisition are more difficult to examine and thus less studied. Identification of factors associated with community‐level risk has the potential to inform structural interventions for HIV prevention and optimize individual‐level interventions within communities.

The risk of HIV transmission through sex is negligible when people with HIV are on antiretroviral therapy (ART) with undetectable viral loads (i.e. “undetectable equals untransmittable, U = U”) [2, 3]. Ecological studies show a strong association between population‐level ART coverage and HIV acquisition rates with good temporal associations between ART scale‐up and declines in HIV incidence [4]. Therefore, the HPTN 071 PopART study tested an intervention aimed to increase ART coverage and thereby reduce the proportion of individuals with unsuppressed HIV at the community level. The intervention goal was to reduce HIV transmission within the study communities [5], consistent with the current UNAIDS 95‐95‐95 goals. The PopART study was conducted in 21 communities—12 in Zambia and nine in South Africa—and measured both viral suppression among people living with HIV and the rate of new acquisitions. The primary result of the trial showed the PopART intervention (Arms A and B combined) achieved a 20% reduction in HIV incidence compared to the standard of care [6].

Here, we leveraged data from the PopART trial to identify community‐level predictors of HIV incidence across the 21 study communities. We examined the association of HIV incidence with community‐level measures reflecting the demographic, sexual behaviour and socio‐economic make‐up of each community. We also assessed the association between prevalence of detectable virus (PDV) and HIV incidence. Lastly, for a more comprehensive understanding of HIV risk during the implementation of the PopART intervention, we evaluated individual‐level factors associated with HIV risk.

## 2 METHODS

The PopART trial was conducted between 2013 and 2018 using a three‐arm triplet‐matched randomized design. The primary goal was to compare HIV incidence among the three study arms: Arm A, universal testing with universal ART; Arm B, universal testing with ART provided according to local guidelines (universal ART beginning in 2016); and Arm C, standard of care. Details of the PopART intervention have been described previously [5]; briefly, pairs of community HIV care providers (CHiPs) went door‐to‐door throughout the community offering HIV information, HIV rapid testing, screening for tuberculosis and sexually transmitted infections, condoms, referral for ART for people living with HIV who were not yet taking ART and voluntary medical male circumcision for uncircumcised individuals without HIV.

To measure the impact of the intervention, the trial enrolled research participants in a population cohort consisting of one randomly selected adult 18–44 years of age recruited from a random sample of ∼ 2000 households in each community [5].

Each enrolled participant had a baseline visit (labelled PC0) and three annual follow‐up visits (labelled PC12, PC24 and PC36). Annual study visits included, but were not limited to, an offer of rapid HIV testing; a collection of plasma for laboratory‐based HIV and viral load testing; and administration of a standardized questionnaire assessing demographic, behavioural and socio‐economic variables.

### 2.1 Variables

The following baseline factors were assessed for association with HIV incidence: socio‐economic factors (education attainment, current employment and household socio‐economic status [SES]), behaviour (alcohol use [AUDIT score] [7] and medical male circumcision) and sexual behaviours (sex partners outside the community [which may reflect the risk of introduction of HIV from elsewhere], pregnancy, ever having sex, having sex in the previous year, number of sexual partners in the previous year and condom use at last sex). A full description can be found in the Table S1.

Community‐level predictors were constructed as means or proportions of individual‐level values, where appropriate, across all participants at baseline for each community and also disaggregated by sex. PDV was defined as the proportion of the entire adult community (those with and without HIV) with a viral load >400 copies/ml.

### 2.2 Laboratory methods

At each visit, a laboratory‐based HIV antigen/antibody test was performed at centralized laboratories in Zambia and South Africa. Additional testing was performed retrospectively at the HPTN Laboratory Center (Johns Hopkins Univ., Baltimore, MD, USA) [6], including confirmation of seroconversion events and determination of the timing of HIV acquisition. Viral load testing was performed using the RealTime HIV‐1 assay (Abbott Molecular; validated dilution method, limit of quantification: 400 copies/ml). Further details of laboratory testing are described elsewhere [6].

### 2.3 Statistical methods

The analyses presented include data from participants in the population cohort enrolled at the start of the trial (PC0). HIV incidence in each community was assessed based on HIV testing at PC12, PC24 and PC36 among participants who were enrolled without HIV. The analyses did not account for missing data due to loss‐to‐follow‐up. Person‐years (PYs) were calculated using the time between visit dates. The midpoint between the last visit without HIV and the first visit with HIV was used as the estimated time of acquisition for seroconversion cases; the time of the first visit with HIV was used as the time of acquisition for cases with acute HIV. Community incidence rates were estimated as the number of new acquisitions divided by the total person time in each community across the entire trial (PC0–PC36).

HIV viral load was measured for all participants with HIV at the PC24 visit; at the PC0, PC12 and PC36 visits, viral load was measured for all seroconverters and ∼75 participants with HIV prevalent infection (non‐seroconverters) selected at random from each community. All participants with HIV were tested at PC24 because it was the midway point of the primary analysis period (PC12–PC36) and ∼75 were chosen at random for the other visits due to budgetary constraints. Inverse probability weighting (accounting for these selection probabilities) within each community was used to estimate community PDV at each visit.

Community‐level models used a two‐stage analysis approach [8]. Briefly, at the first stage, a Poisson regression model for the individual‐level rate of incident acquisition, adjusted for age and sex, was used to estimate the number of expected acquisitions in each community if HIV incidence did not vary by community. A ratio‐residual of observed divided by expected acquisitions was calculated for each community. This estimated the excess risk observed in each community relative to the average risk, adjusted for any differences in sex and age distributions between communities. These ratio‐residuals were then log‐transformed and used as the outcome variable in a linear regression model, and each of the untransformed community‐level variables (except PDV, see below) were assessed as predictors. All linear regression models were adjusted for the study arm, to assess the effect of the community‐level predictors independent of the effect of the study intervention. Adjusted rate ratios (RRs) were obtained from these models which estimated multiplicative change in the community‐level HIV incidence for every 1% additive increase in the community‐level predictor, adjusted for age, sex and study arm. Multivariable models were not assessed due to the small number of communities. This community‐level analysis approach is in concordance with the PopART primary manuscript [9] and a PopART secondary analysis [10].

Sexual risk covariates were assessed “cross‐sex”; that is male (female) community characteristics were assessed as predictors of female (male) HIV risk in this predominantly heterosexual epidemic.

For the analyses of PDV, we used a log‐log linear model, as follows:

ln(ratio−residual)=α+βln(PDV)+e

When β = 1 for this model, we say that incidence is proportional to PDV.

Individual‐level models estimated incident rates and RRs using Poisson regression, overall and in males and females separately. We accounted for clustering by using an indicator for community, making the associations specific to the communities under study. These multivariable models used a hierarchical model selection method [11]: socio‐economic variables were added first, with covariates with a *p*‐value<0.05 retained; next behaviour variables were assessed, again retaining covariates with a *p*‐value<0.05; and finally, sexual behaviour covariates were assessed, and retained using the same threshold. Overall models accounted for age group, sex and their interaction, while sex‐specific models accounted for age group only.

### 2.4 Role of the funding source

The funder had no role in the design and conduct of the study; collection, management, analysis and interpretation of the data; preparation, review or approval of the manuscript; nor decision to submit the manuscript for publication.

### 2.5 Ethical considerations

Participants in the population cohort provided written informed consent, including when ART was initiated outside of the local guidelines, prior to enrolment in the HPTN 071 (PopART) trial. The ethics committees of the University of Zambia, Stellenbosch University and the London School of Hygiene and Tropical Medicine gave approval for this trial.

## 3 RESULTS

### 3.1 Characteristics of the population cohort at baseline

Table 1 shows 38,474 participants were enrolled, with notable differences by sex (27,139 [71%] females; 11,202 [29%] males; 133 missing [<1%]; Table 1). Overall, 7974 (21%) were people living with HIV with a higher prevalence among females than males (25% vs. 11%). Age and sex distributions were similar across communities, except for varying proportions of young persons across triplets (Figure S1).

**Table 1:** Characteristics of the population cohort enrolled at baseline

Demographics			
Age at baseline	Overall	Males	Females
	*N* = 38,474	*N* = 11,202	*N* = 27,139
18–24 years old	15,225 (40%)	4947 (44%)	10,278 (38%)
25–29 years old	8270 (21%)	2239 (20%)	6031 (22%)
30–34 years old	6516 (17%)	1673 (15%)	4843 (18%)
35–39 years old	4735 (12%)	1283 (11%)	3452 (13%)
40–44 years old	3590 (9%)	1059 (9%)	2531 (9%)
Missing	138 (<1%)	1 (<1%)	4 (<1%)

**HIV characteristics** ^a^			
**With HIV test result at baseline**	***N* = 37,006**	***N* = 10,791**	***N* = 26,215**
Without HIV at baseline	29,032 (78%)	9541 (88%)	19,491 (74%)
With HIV at baseline	7974 (22%)	1250 (12%)	6724 (26%)

**Contributing to HIV incidence** ^b^	***N* = 20,765**	***N* = 6433**	***N* = 14,332**
New infections/person‐years (HIV incidence/100 person‐years)	878/59,096 (1.49)	158/18,290 (0.86)	720/40,806 (1.76)
18–24 years old	436/25,407 (1.72)	72/8986 (0.80)	364/16,420 (2.22)
25–29 years old	183/12,148 (1.51)	37/3424 (1.08)	146/8724 (1.67)
30–34 years old	115/9382 (1.23)	21/2426 (0.87)	94/6957 (1.35)
35–39 years old	83/6732 (1.23)	14/1886 (0.74)	69/4847 (1.42)
40–44 years old	61/5427 (1.12)	14/1569 (0.89)	47/3858 (1.22)

**Socio‐economic, behaviour and sexual risk characteristics at baseline** ^a^			
	***N* = 38,336**	***N* = 11,201**	***N* = 27,135**
**Socio‐economic status**			
High SES tertile	11,926 (31%)	3653 (33%)	8273 (31%)
Grade 10 or higher education	22,678 (59%)	7440 (66%)	15,238 (56%)
Full‐time employment	3470 (9%)	1638 (15%)	1832 (7%)
**Behaviour**			
Hazardous or possible dependence for alcohol from the AUDIT score^c^	3517 (9%)	2096 (19%)	1421 (5%)
Individuals reporting medical male circumcision	··	1826 (16%)	··
**Sexual risk**			
Reporting ever having sex	34,604 (90%)	9666 (86%)	24,938 (92%)
Reporting having sex in the last 12 months	26,668 (70%)	7078 (63%)	19,590 (72%)
Reporting sex with two or more partners in the last 12 months	2243 (6%)	1419 (13%)	824 (3%)
Reporting having sex outside the community in the last 12 months	5123 (13%)	1790 (16%)	3333 (12%)
Reported nulligravida females	··	··	5215 (19%)

^a^ Included are participants with non‐missing age and sex at baseline.

^b^ To contribute to HIV incidence, participants must have non‐missing baseline age and sex, and be without HIV at baseline with at least one follow‐up visit with an HIV test result.

^c^ Hazardous or possible dependence for alcohol is an AUDIT score of 8 or more.

HIV incidence was measured for 20,765 participants without HIV enrolled at PC0 with 59,096 PY of follow‐up. There were 878 new acquisitions detected in this cohort (overall incidence 1.49 per 100PY); with 720 acquisitions in females (incidence 1.76 per 100PY) and 158 acquisitions in males (incidence 0.86 per 100PY). The highest HIV incidence among females were aged 18–24 at enrolment (2.22 per 100PY), and among males those aged 25–29 (1.08 per 100PY).

Table 1 also shows other notable differences in baseline characteristics by sex, including males reporting full‐time employment (15% in males vs. 7% in females), hazardous or possible dependence for alcohol (19% in males vs. 5% in females) and having two or more sexual partners in the last 12 months (13% in males vs. 3% in females).

### 3.2 Community‐level socio‐economic, behaviour, sexual behaviour and HIV incidence

Table 2 shows the output for community‐level factors examined for their association with community HIV incidence, including PDV discussed in the next section. Higher community‐level SES was associated with lower community HIV incidence, both overall and for sex‐specific analyses. For every 1% increase in the proportion of individuals in the highest tertile SES in the community, HIV incidence had a relative decrease of 1.5% (adjusted RR: 0.99, 95% confidence interval [CI]: 0.97–1.00, *p* = 0.02) in the community overall, with nearly identical results for cross‐sex analyses (i.e. higher SES in males associated with lower HIV incidence in females, and vice versa).

**Table 2:** Association of community‐level predictors with community‐level HIV incidence, overall and cross‐sex^a^

	HIV incidence overall			HIV incidence in males			HIV incidence in females		
	Proportion overall Mean^b^ (min, max)	Adjusted RR; Adjusted β for PDV (95% CI)^c^	*p*‐value	Proportion in females Mean^b^ (min, max)	Adjusted RR; Adjusted β for PDV (95% CI)^c^	*p*‐value	Proportion in males Mean^b^ (min, max)	Adjusted RR; Adjusted β for PDV (95% CI)^c^	*p*‐value
**Socio‐economic**									
High socio‐economic status	35.0% (4.3, 70)	0.99 (0.97, 1.00)	0.02	34.0% (3.8, 72)	0.99 (0.97, 1.00)	0.02	35.0% (6, 66)	0.99 (0.97, 1.00)	0.02
**Behaviour**									
AUDIT score of hazardous alcohol risk or possible dependence (8+)	10.0% (1.5, 23)	1.01 (0.96, 1.06)	0.72	5.8% (0.8, 15.2)	1.00 (0.92, 1.08)	0.99	21.0% (3.4, 41)	1.02 (0.99, 1.05)	0.24
Medical male circumcision	··	··	··	··	1.00 (0.98, 1.02)	0.83	19.4% (1.9, 41)	1.00 (0.98, 1.02)	0.87
**Sexual behaviour**									
Any sex in the last 12 months	71.5% (44, 90)	1.01 (0.99, 1.04)	0.19	74.3% (46.5, 90)	1.01 (0.98, 1.03)	0.65	64.5% (38, 91)	1.01 (0.99, 1.03)	0.28
Sex with two or more partners in the last 12 months	4.6% (0.8, 12)	1.08 (1.00, 1.17)	0.05	2.9% (0.7, 9.9)	1.04 (0.93, 1.17)	0.45	9.0% (1.2, 16)	1.06 (1.01, 1.12)	0.03
Any sex with a partner outside community	13.9% (4.4, 38)	1.03 (1.00, 1.06)	0.03	12.8% (3.9, 34)	1.01 (0.98, 1.05)	0.48	16.8% (5.2, 49)	1.02 (1.00, 1.05)	0.03
No condom used with last sex	42.2% (21, 67)	0.99 (0.97, 1.01)	0.29	45.9% (22, 72)	0.99 (0.97, 1.01)	0.31	33.0% (19, 51)	0.99 (0.96, 1.01)	0.29
Nulligravida (females only)	··	··	··	20.3% (11.7, 26.5)	0.96 (0.90, 1.01)	0.13	··	0.94 (0.89, 1.00)	0.06

	Proportion overall Mean^b^ (min, max)	Adjusted β (95% CI)^d^	*p*‐value	Proportion in females Mean^b^ (min, max)	Adjusted β (95% CI)^d^	*p*‐value	Proportion in males Mean^b^ (min, max)	Adjusted β (95% CI)^d^	*p*‐value
Prevalence detectable virus at PC0^e^	9.0% (2.2, 13.9)	0.84 (0.48, 1.21)	<0.01	10.2% (2.1, 15.6)	0.55 (0.07, 1.03)	0.03	5.9% (2.0, 12.2)	0.74 (0.19, 1.30)	0.01
Prevalence detectable virus at PC24^e^	6.8% (2.5, 12.0)	0.96 (0.26, 1.26)	<0.01	7.5% (1.9, 12.4)	0.71 (0.31, 1.11)	<0.01	5.9% (2.0, 12.2)	0.75 (0.18, 1.32)	0.01

Abbreviation: RR, rate ratio.

^a^ Across sex describes analyses using predictors calculated from females in the community to predict HIV incidence in males, and vice versa.

^b^ Arithmetic mean of community‐level estimates at PC0.

^c^ Adjusted rate ratio (RR) is the estimated multiplicative change in the community‐level HIV incidence for every 1% additive increase in the community‐level predictor (socio‐economic, behaviour and sexual behaviour predictors), adjusted for age, sex and study arm.

^d^ Adjusted β is the regression coefficient from a log‐log regression model of PDV and HIV incidence, adjusted for age, sex and study arm. One way to interpret this is that halving the community‐level PDV (50% relative reduction in PDV) is estimated to change the HIV incidence by a factor of 2^−β^.

^e^ Proportion of the entire cohort (participants with and without HIV negative) with viral load > 400 copies/ml.

Report of sex partners outside the community was higher for males (16.8%, range 5.2–49) than females (12.8%, range 3.9–34); similarly, more than two partners in the last year was more prevalent in males (9%, range 1%–16%) than in females (3%, range 1%–10%). For both of these sexual risk measures, we saw an association with community HIV incidence. A relative increase of 3% (95% CI: 0%–6%, *p* = 0.03) in community HIV incidence was associated with a 1% increase in the proportion reporting sex outside the community, which was also observed for HIV incidence in females (2% increase, 95% CI: 0%–5%, *p* = 0.03) associated with males reporting sex outside the community, but not for HIV incidence in males for females reporting sex outside the community. A relative increase of 4.6% (95% CI: 0.8%–12%, *p* = 0.05) in community HIV incidence was associated with a 1% increase in the proportion reporting two or more partners, which held for HIV incidence in females (6% increase, 95% CI: 1%–12%, *p* = 0.03) being associated with males reporting more than one partner, but not for males.

There was a moderate negative association between the proportion of nulligravida females in the community and community HIV incidence among females. For every 1% increase in the proportion of females reporting never being pregnant, relative community HIV incidence in females decreased by 5.7% (adjusted RR: 0.94, 95% CI: 0.89–1.00, *p* = 0.06). Other community‐level predictors were not significant (Table S2).

### 3.3 Community‐level PDV and HIV incidence

**Figure 1:**
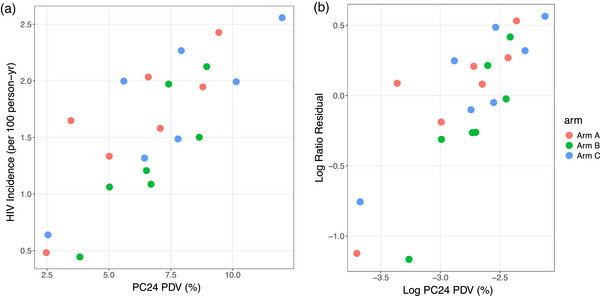
(a) PC24 community prevalence of detectable virus (PDV) and community HIV incidence. (b) Log PC24 community prevalence of detectable virus (PDV) and log ratio residuals.

At PC0, mean PDV across the 21 communities was 9.0% (range 2.2%–13.9%). At PC24, mean PDV decreased to 6.8% (range 2.5%–12.0%). Community PDV at PC0 and PC24 was associated with community HIV incidence. At PC24, we saw an adjusted regression coefficient of 0.96 (95% CI: 0.26, 1.26, *p*<0.01), which says that for every 50% relative reduction in community PDV, there was a 49% (95% CI: 37%–58%) relative decrease in HIV incidence (Table 2 and Figure 1); similar reductions were observed by sex; the relative reduction in HIV incidence in females, as predicted by halving the PDV in males, was 59% (95% CI: 40%–88%, *p* = 0.01), and in males, predicted by halving the PDV in females, was 61% (95% CI: 46%–80%, *p*<0.01).

**Figure 2:**
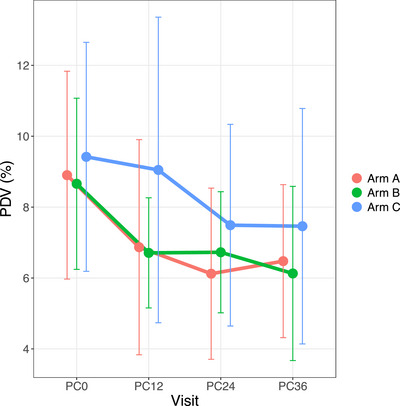
Community prevalence of detectable virus (PDV) over time (averaged by arm with naïve *t*‐intervals).

We observed a decrease in PDV in the two intervention arms (Arms A and B) by PC12; reductions were not seen in the standard of care arm until PC24 (Arm C, Figure 2). By PC24, there was an estimated 15% relative reduction in mean PDV for Arm A and Arm B communities compared to Arm C communities (Figure 2).

### 3.4 Individual‐level socio‐economic, behaviour, sexual behaviour and risk of HIV acquisition

SES was significantly correlated with HIV incidence (Table 3); participants in the lowest tertile of SES had a 51% increased risk of HIV compared to participants in the highest tertile of SES (relative risk: 1.51, 95% CI: 1.24, 1.84, *p*<0.01). This result was more pronounced in males (relative risk: 2.84, 95% CI: 1.71, 4.71) than in females (relative risk: 1.31, 95% CI: 1.05, 1.63). Both low and hazardous risk AUDIT scores were associated with an increase in HIV risk compared to alcohol abstainers (low‐risk AUDIT relative risk: 1.54, 95% CI: 1.23, 1.93, *p*<0.01; hazardous risk AUDIT relative risk: 1.74, 95% CI: 1.31, 2.32, *p*<0.01). This association was similar by sex. Medical male circumcision led to an estimated 45% reduction in HIV risk in males (relative risk: 0.56, 95% CI: 0.32, 0.95, *p* = 0.03) compared to uncircumcised males.

**Table 3:** Individual‐level baseline associations with HIV risk (overall, by males and by females)

	Overall		Males only		Females only	
	Adjusted relative risk^a^ (95% CI)	*p*‐value^b^	Adjusted relative risk^c^ (95% CI)	*p*‐value^b^	Adjusted relative risk^c^ (95% CI)	p‐value^b^
**Socio‐economic**						
**Socio‐economic status**		**<0.01**		**<0.01**		**<0.01**
Highest tertile	1		1		1	
Middle tertile	1.14 (0.95, 1.38)	0.16	2.32 (1.44, 3.75)	<0.01	0.98 (0.81, 1.21)	0.90
Lowest tertile	1.51 (1.24, 1.84)	<0.01	2.84 (1.71, 4.71)	<0.01	1.31 (1.05, 1.63)	0.01
**Behaviour**						
**AUDIT score** ^d^		**<0.01**		**0.06**		**<0.01**
Abstainer	1		1		1	
Low risk	1.54 (1.23, 1.93)	<0.01	1.55 (0.97, 2.48)	0.06	1.46 (1.13, 1.88)	<0.01
Hazardous consumption	1.74 (1.31, 2.32)	<0.01	1.95 (1.22, 3.14)	<0.01	1.70 (1.19, 2.41)	<0.01
Possible dependence	1.35 (0.88, 2.08)	0.16	1.07 (0.54, 2.14)	0.84	1.82 (1.08, 3.05)	0.02
**Male circumcision**				**0.03**		
None	··	··	1		··	··
Medical	··	··	0.55 (0.32, 0.95)	0.03	··	··
Traditional	··	··	0.72 (0.43, 1.22)	0.21	··	··
**Sexual behaviour**						
**Any sex ever (ref. No)**	1.84 (1.34, 2.52)	**<0.01**	NA^e^	NA	2.21 (1.52, 3.21)	**<0.01**
**Any sex in the last 12 months (ref. No)**	0.61 (0.51, 0.73)	**<0.01**	NA	NA	0.59 (0.48, 0.71)	**<0.01**
**Sex with two or more partners in the last 12 months (ref. No)**	1.82 (1.38, 2.39)	**<0.01**	NA	NA	2.25 (1.64, 3.09)	**<0.01**
**Any sex with a partner outside community (ref. No)**	1.28 (1.04, 1.59)	**0.02**	NA	NA	1.31 (1.04, 1.65)	**0.02**

^a^ Adjusted for community, age, sex and all other hierarchical model predictors.

^c^ Adjusted for community, age and all other hierarchical model predictors.

^b^ Bolded *p*‐values are likelihood ratio test *p*‐values when predictors have more than one categorical level.

^d^ AUDIT scores: Abstainer = 0, Low risk = 1–7, Hazardous consumption = 8–14, Possible dependence = 14+.

^e^ Not applicable: Missing men's output is due to the dropping of non‐significant predictors in the hierarchical model for men.

Participants ever having sex at baseline had an estimated 84% increased risk of acquiring HIV compared to those never having sex (relative risk: 1.84, 95% CI: 1.34, 2.52, *p*<0.01). Sexually active participants (sex in the last year) had a 39% lower risk of HIV compared to those not sexually active at baseline (relative risk: 0.61, 95% CI: 0.51, 0.73, *p*<0.01); while those reporting two or more sexual partners in the last year had an 81% increase in HIV risk compared to those reporting zero or one partner (relative risk: 1.81, 95% CI: 1.38, 2.39, *p*<0.01). Individuals reporting sex partners outside the community had 28% higher HIV risk compared to those who did not (relative risk: 1.28, 95% CI: 1.04, 1.59, *p* = 0.02). While the directions of the effects were the same, these three sexual risk predictors were not statistically significant in male participants but were significant in female participants.

## 4 DISCUSSION

The HPTN 071 (PopART) trial provided a unique opportunity to directly measure community‐level risk factors associated with HIV incidence. While these data are from 2013 to 2018, in the current HIV epidemic where treatment coverage (both PrEP and ART) is increasing yet HIV incidence remains high in certain areas, the community‐level predictors evaluated here could be particularly helpful in identifying communities with persistently high HIV incidence where interventions should be directed.

We found several community‐level factors were associated with increased HIV incidence, including less wealth, having sex partners outside the community and having a higher number of sex partners (compared to fewer). The strongest association with decreased community HIV incidence observed in our study was higher community‐level SES (compared to lower SES), an association that was consistent for males and females. These SES results support the previous findings of Santelli et al. [12] which found that higher SES groups showed greater declines in HIV incidence from 1997 to 2018 in rural Uganda. More recently, the COVID‐19 pandemic has widened wealth inequality around the world, including in sub‐Saharan Africa [13, 14], and our research adds to growing calls for structural anti‐poverty measures to improve population health [15].

The Four Cities study looked at population‐level risk factors for HIV prevalence and identified metrics, such as average age of first marriage, circumcision prevalence and population prevalence of herpes simplex virus type 2 (HSV‐2) [16]. Other ecological studies have found associations between HIV prevalence and the time between sexual debut and marriage [17] and population‐level sexual risk behaviour [18, 19, 20]. We found modest associations between community HIV incidence and two community‐level measures of sexual risk in females: (1) lower HIV incidence among females was associated with an increased proportion of nulligravida females, and (2) higher HIV incidence among females was associated with an increased proportion of males who had sex partners outside the community. Prevalence of detectable virus (PDV), both at baseline and 2 years after the implementation of the PopART intervention, was a strong predictor of HIV incidence across these communities. PDV decreased in all three study arms, with a more rapid decrease and a lower mean PDV in the two intervention arms (Arms A and B) compared to the standard of care arm (Arm C); suggesting that decreased PDV mediated the observed intervention effect on HIV incidence. The differences achieved in PDV by the study arm at PC24 varied by triplet (Figure S2), suggesting that this measure could be useful for monitoring the effectiveness of strategies, such as universal testing and treatment, to reduce HIV incidence.

Kelley and colleagues first proposed the concept of using PDV in a study of men who have sex with men in the United States to capture transmission risk and inequalities in healthcare coverage [21]. This measure was used in high‐prevalence areas to identify transmission hotspots [22] and within key population networks [23, 24]. Two studies demonstrated its superiority over other metrics to predict HIV incidence [25, 26]. Both studies are ecological studies and, therefore, have the well‐recognized limitation of not being able to demonstrate causation.

Our study is the first to use PDV in a randomized trial where differences in PDV and measured HIV incidence can be assumed to be due to the intervention applied compared to the standard of care. This direct demonstration of the association between universal testing and treatment and PDV, with the associated reduction in HIV incidence, provides a powerful metric for assessing the effectiveness of HIV prevention interventions without having to measure longitudinal HIV incidence, a challenging and costly undertaking due to the requirements of following large cohorts over time.

At the individual level, moderate‐to‐strong associations were observed between HIV incidence and reported SES, AUDIT score categories, medical male circumcision and sex history in the last year. Our finding of a 45% reduction in HIV risk for medically circumcised men compared to uncircumcised men aligns with previously published findings [27, 28, 29]. However, contrasting to Kong et al. [30], this finding did not translate to the community level due to several communities having both high proportions of medical male circumcision and high HIV incidence. Those reporting sex in the last year showing a decrease in HIV risk compared to those who did not report sex in the last year is a surprising finding. While we have not found other published research with this finding, it may be possible that those not reporting sex in the last year at baseline (including those who had sexual debut during PopART) had less bargaining power during future sexual acts and may have engaged in riskier sexual behaviour.

Our study has some limitations. The community‐level models did not include multivariate adjustments because of the relatively small number of communities. Analyses were unweighted, reflecting measures and incidence specific to the participants in the population cohort rather than the entire community with the main imbalance being the proportion of females enrolled in the population cohort. However, we did perform analyses that separated males and females in addition to the overall analyses. Few sexual behaviour covariates were measured in the whole cohort; these may not have been sufficiently sensitive to measure the risk of HIV exposure at the community level. We also acknowledge the sensitive nature of some aspects of the questionnaire and there may be bias in how participants answered. Approximately 30% of participants enrolled at baseline did not have a follow‐up visit with an HIV test result during the study period and thus their data were censored. Although we cannot rule out selection bias, there was little evidence retention differed by trial arm, demographic or behaviour risk factors, as examined in Table S3.

## 5 CONCLUSIONS

In this population cohort, followed for 3 years during the implementation of the PopArt intervention, we found several community‐level factors were associated with increased HIV incidence, including less wealth, having sex partners outside the community and having two or more sexual partners. Identification of these factors associated with community‐level HIV incidence can help target high‐incidence communities and help to inform future structural interventions for HIV prevention within those communities. In addition, community PDV was highly predictive of HIV incidence supporting the use of PDV as a useful measure to assess the effectiveness of community‐level interventions to reduce HIV incidence.

## COMPETING INTERESTS

SE reports grant funding from the NIH, and the study team reports grant funding from the NIH, the international initiative for impact evaluation (3ie), the U.S. President's Emergency Plan for AIDS Relief (PEPFAR) and the Bill and Melinda Gates foundation. SF reports membership on the Gilead Scientific advisory board, DSMB CORE HIV vaccine trial (unpaid), DSMB CHAPS EDCTP (unpaid) and the IAS cure scientific advisory board (unpaid). HA reports membership in the technical review panel for the Global Fund.

## AUTHORS’ CONTRIBUTIONS

**TS**: Statistical analysis, data interpretation and writing. **HA**: Conceptualization, study implementation, study design, data curation and writing. **PB**: Study design, data collection and manuscript review. **JB**: Study design, data collection and manuscript review. **KS**: Manuscript review. **NK**: Manuscript review. **MD**: Manuscript review. **BK**: Data collection, lab testing and manuscript review. **SF**: Study design, statistical analysis review and manuscript review. **EW**: Data curation, data verification and manuscript review. **AM**: Conceptualization, project administration & supervision, and manuscript review. **SE**: Laboratory lead, lab testing, data curation and manuscript review. **SF**: Conceptualization, study design, study implementation and manuscript review. **RH**: Conceptualization, study design, study implementation, statistical analysis review and manuscript review. **DD**: Study design, study implementation, data curation, statistical analysis review, data interpretation and writing.

## FUNDING

This work was supported by funding from the Bill and Melinda Gates Foundation through the INPUTT project. HPTN 071 (PopART) was sponsored by the HIV Prevention Trials Network (HPTN) and supported by the National Institute of Allergy and Infectious Diseases (NIAID), with funding from the U.S. President's Emergency Plan for AIDS Relief (PEPFAR); the International Initiative for Impact Evaluation (3ie) with support from the Bill and Melinda Gates Foundation; as well as the National Institute on Drug Abuse (NIDA) and the National Institute of Mental Health (NIMH), both part of the National Institutes of Health (NIH).

## DISCLAIMER

The funder had no role in the design and conduct of the study; collection, management, analysis and interpretation of the data; preparation, review or approval of the manuscript; and decision to submit the manuscript for publication.

## Supporting information


**Table S1**: Covariates and the measures used.
**Table S2**: Extended Community‐Level Demographics and Sexual Risk Output.
**Table S3**: Loss to Follow‐Up among HIV Incidence Analysis Cohort by Baseline Characteristics.
**Figure S1**: Age‐Sex Distributions of the Population Cohort at Baseline, by Community.
**Figure S2**: Community PC24 Prevalence of Detectable Virus (PDV) by Study Arm, Triplet.

## Data Availability

Data used in this study are available upon request, with no end date. This includes de‐identified participant data with a data dictionary. Requests can be sent to HPTN-Data-Access@scharp.org. The study protocol is available here: https://pubmed.ncbi.nlm.nih.gov/24524229/.
