# Supplementary material for: Community‐ and individual‐level correlates of HIV incidence in HPTN 071 (PopART)
Source: J Int AIDS Soc. 2023 Aug 29;26(8):e26155. doi: 10.1002/jia2.26155 (PMC10465013; doi:10.1002/jia2.26155)
Supplement: Supplementary file 1 — Table S1: Covariates and the measures used. Table S2: Extended Community‐Level Demographics and Sexual Risk Output. Table S3: Loss to Follow‐Up among HIV Incidence Analysis Cohort by Baseline Characteristics. Figure S1: Age‐Sex Distributions of the Population Cohort at Baseline, by Community. Figure S2: Community PC24 Prevalence of Detectable Virus (PDV) by Study Arm, Triplet. [file JIA2-26-e26155-s001.docx]

**Supplementary Table 1: Covariates and the measures used**

| Covariate | Measure |
| --- | --- |
| **Socio-economic** | |
| Socio-economic status | First principal component value using the Wealth Index variables, calculated by Country. Tertiles were also created (Low/Mid/High) within country. See below for documentation. |
| Education | Completed grade 10 or higher? (Yes/No) |
| Employment | Employed (Yes/No) as well as type of employment (Full-time, Part-time, etc). |
| **Behavior** | |
| AUDIT score | Sum of AUDIT measures, transformed into a binary measure of risk (8+ AUDIT score) or using raw score |
| Male circumcision | Medical, Traditional, or Not Circumcised |
| **Sexual Risk** | |
| Pregnancy | Number of pregnancies (0 vs 1 or more) |
| Sexual history | Have you ever had sex? (Yes/No)  Have you had sex in the last 12 months? (Yes/No)  Did you have two or more sexual partners in the last 12 months? (Yes/No)  Have you had a sexual partner outside the community in the last 12 months? (Yes/No) |
| Condom use at last sex act | Did you use a condom during your last sex act? (Yes/No) |
| **Circulating Virus (Community-Level Only)** | |
| Prevalence of Detectable Virus (PDV) | Number of HIV-positive individuals who are virally unsuppressed, using a cutoff of 400 copies/mL, divided by the total individuals in the community (both HIV-negative and HIV-positive) |

**Supplementary Figure 1: Age-Sex Distributions of the Population Cohort at Baseline, by Community**


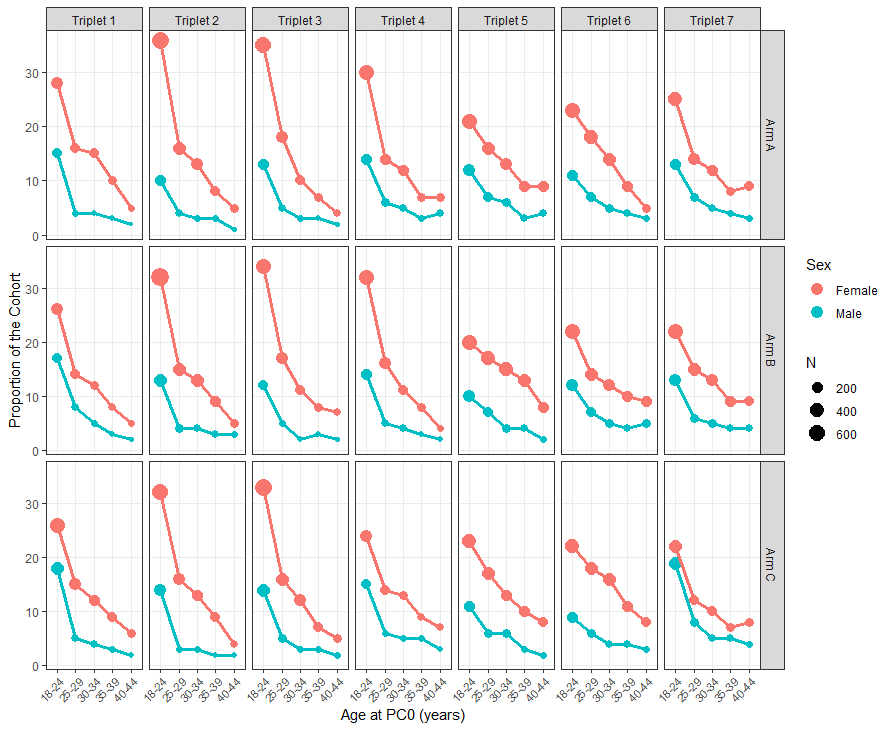


**Supplementary Figure 2: Community PC24 Prevalence of Detectable Virus (PDV) by Study Arm, Triplet**


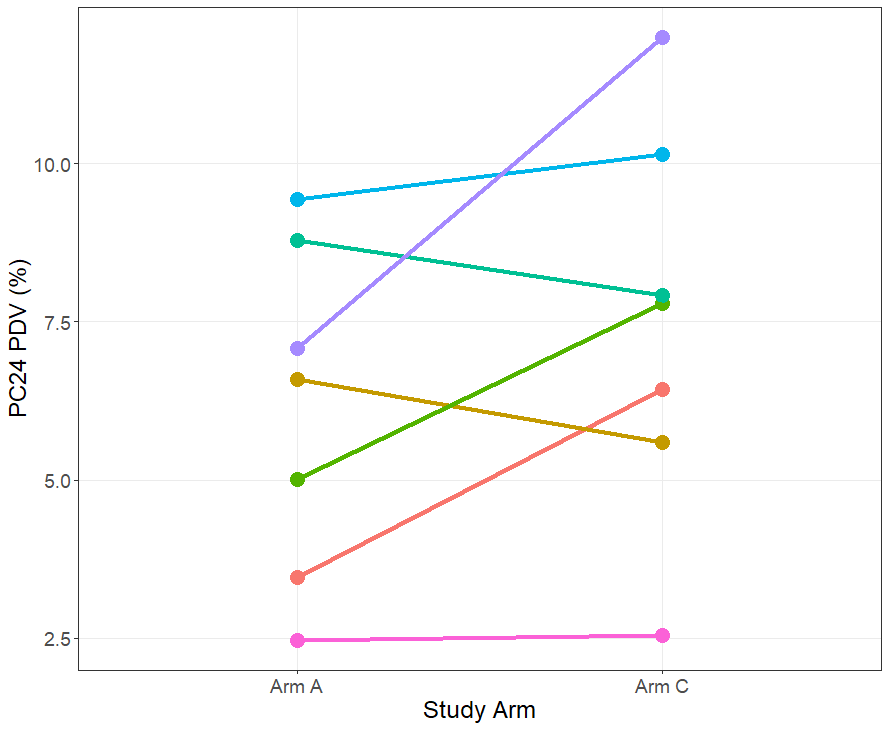

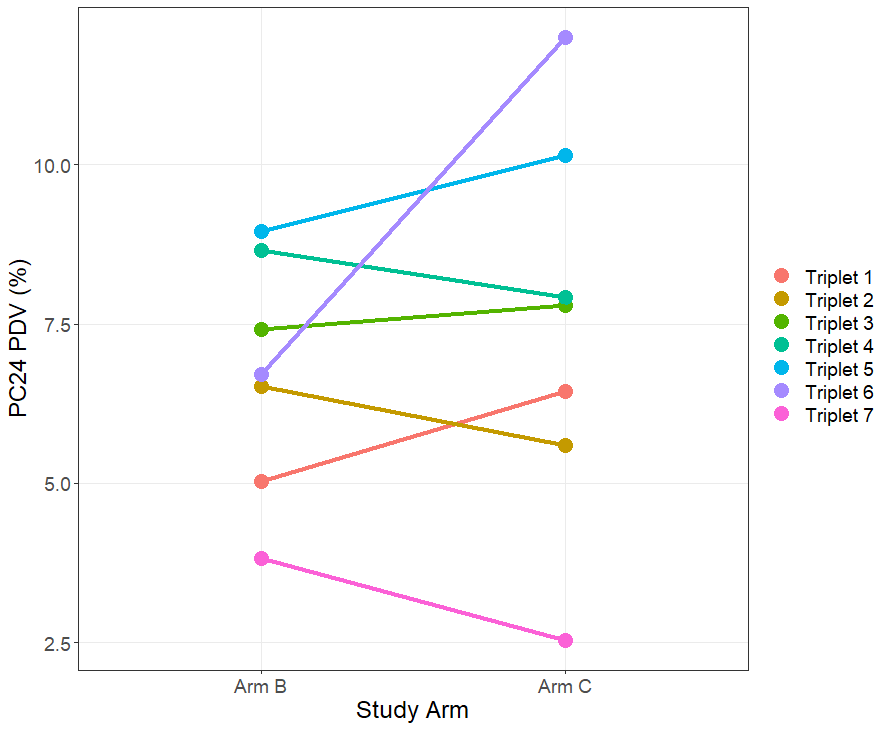


**Supplementary Table 2: Extended Community-Level Demographics and Sexual Risk Output**

| Factor (Subset) | HIV Incidence Subset | Adjusted RR (95% CI) | P-Value |
| --- | --- | --- | --- |
| **Socio-economic** | | | |
| SES – Prop’n High (All) | **All** | 0·99  (0·97, 1·00) | 0·02 |
| SES – Prop’n High (Females) | **Males** | 0·99  (0·97, 1·00) | 0·02 |
| SES – Prop’n High (Males) | **Females** | 0·99  (0·97, 1·00) | 0·02 |
|  | | | |
| Education – Prop’n Grade 10+ (All) | **All** | 1·00  (0·99, 1·01) | 0·99 |
| Education – Prop’n Grade 10+ (Females) | **Males** | 1·00  (0·99, 1·01) | 0·39 |
| Education – Prop’n Grade 10+ (Males) | **Females** | 1·00  (0·99, 1·02) | 0·58 |
|  | | | |
| Employment – Prop’n Unemployed (All) | **All** | 1·03  (0·99, 1·06) | 0·11 |
| Employment – Prop’n Unemployed (Females) | **Males** | 1·02  (0·99, 1·06) | 0·12 |
| Employment – Prop’n Unemployed (Males) | **Females** | 1·01  (0·97, 1·04) | 0·72 |
| **Behavior** | | | |
| AUDIT – Prop’n Score 8+ (All) | **All** | 1·01  (0·96, 1·06) | 0·72 |
| AUDIT – Prop’n Score 8+ (Females) | **Males** | 1·00  (0·92, 1·08) | 0·99 |
| AUDIT – Prop’n Score 8+ (Males) | **Females** | 1·02  (0·99, 1·05) | 0·24 |
|  | | | |
| Circumcision – Prop’n Medical (Males) | **All** | 1·00  (0·98, 1·02) | 0·93 |
| Circumcision – Prop’n Medical (Males) | **Males** | 1·00  (0·98, 1·02) | 0·83 |
| Circumcision – Prop’n Medical (Males) | **Females** | 1·00  (0·98, 1·02) | 0·87 |
| **Sexual Behavior** | | | |
| Sex in Last 12 mo. – Prop’n (All) | **All** | 1·01  (0·99, 1·04) | 0·19 |
| Sex in Last 12 mo. – Prop’n (Females) | **Males** | 1·01  (0·98, 1·03) | 0·65 |
| Sex in Last 12 mo. – Prop’n (Males) | **Females** | 1·01  (0·99, 1·03) | 0·28 |
|  | | | |
| Sex with 2 or more partners in the last 12 months (All) | **All** | 1·08  (1·00, 1·17) | 0·05 |
| Sex with 2 or more partners in the last 12 months (Females) | **Males** | 1·04  (0·93, 1·17) | 0·45 |
| Sex with 2 or more partners in the last 12 months (Males) | **Females** | 1·06  (1·01, 1·12) | 0·03 |
|  | | | |
| Sex Outside Comm. (All) | **All** | 1·03  (1·00, 1·06) | 0·03 |
| Sex Outside Comm. (Females) | **Males** | 1·01  (0·98, 1·05) | 0·48 |
| Sex Outside Comm. (Males) | **Females** | 1·02  (1·00, 1·05) | 0·03 |
|  | | | |
| Condom at Last Sex – Prop’n No (All) | **All** | 0·99  (0·97, 1·01) | 0·29 |
| Condom at Last Sex – Prop’n No (Females) | **Males** | 0·99  (0·97, 1·01) | 0·31 |
| Condom at Last Sex – Prop’n No (Males) | **Females** | 0·99  (0·96, 1·01) | 0·29 |
|  | | | |
| Pregnancy – Prop’n Never Pregnant (Females) | **All** | ·· | ·· |
| Pregnancy – Prop’n Never Pregnant (Females) | **Males** | 0·96  (0·90, 1·01) | 0·13 |
| Pregnancy – Prop’n Never Pregnant (Females) | **Females** | 0·94  (0·89, 1·00) | 0·06 |

**Supplementary Table 3: Loss to Follow-Up among HIV Incidence Analysis Cohort by Baseline Characteristics**^†^

|  | No follow-up HIV test result |
| --- | --- |
| **Intervention Arm^‡^** |  |
| Arm A | 3005/9569 (31%) |
| Arm B | 2915/10203 (29%) |
| Arm C | 2639/9260 (28%) |
| **HIV Status** | |
| With HIV | 2620/7974 (33%) |
| Without HIV | 8559/29032 (29%) |
| **Sex** | |
| Male | 3193/9541 (33%) |
| Female | 5366/19491 (28%) |
| **Age** | |
| 18-24 years old | 4291/13254 (32%) |
| 25-29 years old | 1932/6157 (31%) |
| 30-34 years old | 1144/4330 (26%) |
| 35-39 years old | 678/2951 (23%) |
| 40-44 years old | 514/2340 (22%) |
| **SES** | |
| Lowest Tertile | 3012/8670 (35%) |
| Middle Tertile | 2496/8925 (28%) |
| Highest Tertile | 2444/9671 (25%) |
| **Audit Score** | |
| Audit Score of 0 | 6202/20680 (30%) |
| Audit Score of 1-7 | 939/3463 (27%) |
| Audit Score of 8+ | 804/2585 (31%) |
| **Sex in the last 12 months?** | |
| Yes | 5878/19941 (29%) |
| No | 2494/8308 (30%) |
| **Sex with 2 or more partners**  **in the last 12 months?** | |
| Yes | 587/1732 (34%) |
| No | 5116/17602 (29%) |
| **Sex Outside the Community**  **in the last 12 months?** | |
| Yes | 1273/3778 (34%) |
| No | 6969/23955 (29%) |
| **Condom used at Last Sex?** | |
| Yes | 4735/15603 (30%) |
| No | 3501/12134 (29%) |
|  | |

^†^ To be included in the HIV Incidence Analysis Cohort, participants must have non-missing baseline age and sex, and (for characteristics other than baseline HIV status) be without HIV at baseline.

^‡^ In an additional analysis using multivariate logistic regression, after accounting for age and sex the average probability of loss-to-follow-up was 29.5% for each intervention arm.

**Details of the multi-level analyses**

1. **Community-level Analyses**

We use a two-stage analysis approach for cluster randomized trials. In the first stage of analysis, an individual-level Poisson regression model is constructed with covariates of age group (*a*), sex (*s*), and the age-by-sex interaction while using log-person-years as an offset. Since the age of a participant changes throughout the trial and age is correlated with HIV risk, we use a Lexis Expansion of age in these analyses using each participant’s known or estimated birthdate. That is, a participant can contribute different amounts of person-time (time interval *k* below) to different age groups. Note that arm and any community-level predictor of interest is omitted from the covariates at this stage.

log($E[Y_{ik}]$) = log(*offset_k_*) + $\beta_{0}$ + $\beta_{1}a_{ik}$ + $\beta_{2}s_{ik}$ + $\beta_{3}a_{ik}s_{ik}$

*i = 1, 2, … individual*

*k = time interval for person i*

To clarify, “time interval *k*” refers to the fact that each participant’s person-time is split using the Lexis Expansion according to their birthday, such that each person contributes one year of person-time per their age in the study, with less than or equal to one year of person-time at their first and last time intervals in the study. For example, a participant can enroll in the study at age 23 and exit the study at age 26. Their observations would be split up (according to their birthday) they contribute <=1 year of person-time to age 23 (time from enrollment until 24^th^ birthday), one year of person-time to each age 24 and 25, and <=1 year of person-time to age 26 (from 26^th^ birthday to last visit date in the study). Thus they contribute some person-time to age group 18-24 years old and the rest of their person-time to age group 25-29 years old.

Predictions from the model (1) are summarized up to the community-level as the predicted number of events for a given community. A ratio residual (*RR*) of observed events to predicted events for each community is calculated and log-transformed (to reduce skewness in stage two of the analysis).

*log(RR) = log(*${observed events}/{predicted events}$*)*

In stage two of this analysis, we use multiple linear regression of the log-ratio-residuals on intervention group ($g_{j})$and the community-level predictor of interest ($x_{tj})$. Note that when examining PDV, we used a log-log model; that is, log-transformed both the response and the PDV predictor. Intervention group had a significant effect on HIV incidence and therefore we include it in the modelling.

log(RR_tj_) = $\beta_{0}$ + $\beta_{1}g_{j}$ + $\beta_{2}x_{tj}$

*t = triplet 1, 2, …, 7*

*j = group A, B, C*

Baseline (PC0) community-level predictors are constructed as either a mean or proportion from the individuals within each community. Measures of circulating virus (viral burden and viral suppression) are proportions from individuals within each community at different time points in the study (PC0 and PC24). These variables may be separated by sex depending upon the scientific questions of interest.

1. **Individual-level Analyses**

We use Poisson regression to assess baseline (PC0) individual-level predictors of HIV incidence in a hierarchical model selection method, while accounting for community ($c$), age-group at baseline ($a$), sex ($s$), and the age-by-sex interaction. The response is HIV infection in the trial. Each predictor ($x_{*ij}$) is an individual-level baseline variable of interest, which can be quantitative or categorical. The *offset* is the time for that participant in the trial. When we have sex-specific analyses, there is no adjustment for sex in the model.

log(${E[Y}_{ij}]$) = log(*offset*) + $\beta_{0}$ + $\beta_{1}c_{i}$ + $\beta_{2}a_{ij}$ + $\beta_{3}s_{ij}$ + $\beta_{4}a_{ij}s_{ij}$ + $\beta_{5}x_{1ij}$ + $\beta_{6}x_{2ij}$ + …

*i = 1, 2, …, 21 community*

*j = 1, 2, … individual*

We first build a model with socio-demographic variables and keep any covariates with a p-value < 0.05; next adding socio-economic and socio-behavioral variables and keep those covariates with a p-value < 0.05; and finally adding sexual behavioral covariates and doing the same model selection for those covariates. The final multivariate model is then constructed using all covariates that passed this hierarchical model selection procedure.

**How were the Socio-economic status (SES) tertiles created?**

This country-specific variable was created by running a principal component analysis (PCA) on a set of variables that are recoded in binary (0/1) format. The pool of variables for the PCA included:

1. Access to a working cellphone (0=no, 1=yes)
2. Access to a bicycle (0=no, 1=yes)
3. Access to a car/bakkie (0=no, 1=yes)
4. Access to electricity to house (0=no, 1=yes)
5. Access to a TV (0=no, 1=yes)
6. Access to a fridge/freezer (0=no, 1=yes)
7. Access to a computer/laptop (0=no, 1=yes)
8. Access to a CD/MP3 player (0=no, 1=yes)
9. Improved sanitation source

- 0 if one of the following:

Shared VIP latrine, Pail/bucket, Communal chemical latrine, Bush, Other

- 1 if one of the following:

Flush toilet, Shared flush toilet, Own pit latrine, Shared pit latrine, Own VIP latrine

1. Improved flooring

- 0 if one of the following:

Dirt/earth, Wood/plank, Other

- 1 if one of the following:

Parquet, Lino, Cement, Tile

1. Improved drinking water source

- 0 if one of the following:

Unprotected/shallow well river/dam/lake/pan, Bowser/tanker, Other

- 1 if one of the following:

Piped indoors, Stand pipe/tap within plot, Communal tap, Borehole, Protected well

1. Improved cooking source

- 0 if one of the following:

Gas, Paraffin, Charcoal, Wood, Other

- 1 if one of the following:

No cooking done in household, Electricity (mains), Electricity (individual solar)

1. Improved building structure of home

- 0 if one of the following:

Caravan/tent, Worker's hostel, Shack, Other

- 1 if one of the following:

Single unit/brick structure on its own stand, Cluster/multi-unit, Traditional hut/structure made from traditional material, Flat in block of flats, Servant quarters

Due to lack of variation, some of the binary measures were excluded from the PCA, at a country-specific level. Zambia included all variables except improved drinking water source and improved cooking source, while South Africa included all 13 measures. Applying PCA separately to both countries, we used the first principal component to develop a standardized score, and then grouped individuals according to the tertile that their score fell in. Thus, the final variable analyzed was a 3-category country-specific wealth index: Lowest Tertile, Middle Tertile, and Highest Tertile.
